# Fecal Secretory Immunoglobulin A and Lactate Level as a Biomarker of Mucosal Immune Dysfunction in Horses With Colic

**DOI:** 10.1111/jvim.70073

**Published:** 2025-03-27

**Authors:** Agnieszka Żak‐Bochenek, Zuzana Drábková, Vaiva Sergedaite, Natalia Siwińska, Joanna Bajzert, Dominika Pasak, Anna Chełmońska‐Soyta

**Affiliations:** ^1^ Department of Immunology, Pathophysiology and Veterinary Preventive Medicine Wroclaw University of Environmental and Life Sciences Wroclaw Poland; ^2^ Equine Clinic, Faculty of Veterinary Medicine University of Veterinary Sciences Brno Brno Czech Republic; ^3^ Department of Internal Diseases With Clinic of Horses, Dogs and Cats Wroclaw University of Environmental and Life Sciences Wroclaw Poland

**Keywords:** abdomen, horses, intestine, lactates

## Abstract

**Background:**

Colic‐related obstructions can reduced intestinal mucosa function and cause dysbiosis in horses, but it is unclear how defense barrier and secretory immunoglobulin A (SIgA) secretion is disrupted.

**Objectives:**

The aim of the study is to evaluate the effect of severity of colic signs and treatments on fecal SIgA and fecal lactate in horses.

**Animals:**

Sixty‐two client owned hospitalised horses with colic and eight healthy horses.

**Methods:**

Prospective clinical trial. Fecal samples were taken daily for 7 days. SIgA was analyzed using ELISA, and D/L‐lactate measured with a commercial kit.

**Results:**

At Day 0, SIgA values in the colic medical and colic surgical groups were significantly higher than in the control stable group (*U* = 126.0, *p* = 0.099, Cliff's ∆ = 0.58 and *U* = 248.0, *p* = 0.005, Cliff's ∆ = 0.72, respectively). We found significant correlation between fecal SIgA and fecal lactate level in D0 (*r*_s_ = 0.421, *p* = 0.038).

**Conclusions:**

This study demonstrates the feasibility of using fecal samples to identify biomarkers of colic in horses. An increase in fecal SIgA in horses with colic might suggest the presence of inflammation within the intestines and disruption of the mucosal barrier. These data highlight changes in gastrointestinal barrier and immune function and the intestinal microbiota's metabolic activity in horses with colic.

**Abbreviations**
IECs: intestinal epithelial cells
NSAIDs: nonsteroidal anti‐inflammatory drugs
SIgA: secretory immunoglobulin A
WBC: white blood cells

## 1 Introduction

Horses as a species are notable susceptibility to colic which presents with varying degrees of severity within the digestive system, encompassing conditions such as simple obstruction, strangulating obstruction, and severe inflammatory processes, among others. Any of the aforementioned diseases, like simple or strangulating obstruction, can lead to ischemic changes within the intestine [1, 2]. Ischemic conditions, lumen obstruction by fecal masses, and the initiation of anaerobic metabolism of intestinal bacterial flora occurring during the colic episode contribute to severe damage to the intestinal mucosa [1, 2].

Colic is linked with alterations in the microbiome, dysbiosis, and disruptions in the production of volatile fatty acids, lactic acid included [3, 4]. D‐lactate results from bacterial fermentation, whereas, L‐lactate is synthesized by mammalian cells under conditions of oxygen deprivation, as products of anaerobic glycolysis [5]. An increase in total lactate concentrations in both serum and peritoneal fluid might correlate with ischemic intestinal disorders during colic, mostly with strangulating obstruction [5].

The continuity of mucosal cells, their close adhesion, and the secretion of secretory immunoglobulin A (SIgA) by intestinal epithelial cells (IECs) form the basis of nonspecific mucosal immunity mechanisms [1].

However, the effect of colic on IECs disruption and on the transport of SIgA, which is essential to maintain mucosal homeostasis, remains unknown. Investigations on the effects of intestinal ischemia–reperfusion disorders on IgA secretion have been conducted in rats and mice, where intestinal ischemia was experimentally induced, demonstrating contradictory outcomes (either an increase in serum IgA or a decrease in SIgA within the intestinal lumen) [6, 7].

In human and small animal medicine, fecal SIgA serves as an indicator of gut secretory immunity and barrier function; nevertheless, its applicability in horses remains unexplored [8, 9]. To date, in horses, fecal SIgA analysis has been examined in stress‐related studies and for assessing its correlation with hospitalization [10, 11]. The D‐ and L‐lactate content of equine feces has not yet been evaluated in the context of colic and its progression.

The aim of this study is to ascertain changes in the concentration of fecal SIgA in horses exhibiting signs of colic of varying severity and subjected to either conservative or surgical treatment modalities, including their dynamics during recuperation. A further objective of the study was to evaluate the prevalence of microbiome‐related metabolic disorders via fecal lactate concentration.

## 2 Materials and Methods

Prospective clinical trial.

### 2.1 Animals

The study material comprised fecal samples obtained from equine patients at the Equine Clinic of University of Veterinary Sciences in Brno, Czech Republic, between August and December 2023, with prior informed consent from the owners. The Ethics Committee of the University of Veterinary Sciences Brno sanctioned the study under protocol number: ES 2‐2024. The study included horses referred to the clinic due to colic signs (52 horses total). Each horse underwent standard diagnostic procedures upon admission, including a clinical examination, blood tests (hematology, acid–base balance, lactate), rectal exams, transabdominal ultrasounds, abdominocentesis, and nasogastric intubation. Based on diagnostic outcomes, horses were designated either for surgical intervention (ventral midline laparotomy—comprising 16 horses within the “colic surgical” group) or subjected to conservative treatment—involving 36 horses in the “colic medical” group. Prior to clinic admission, a majority of the horses received analgesics and spasmolytic, treatments that were maintained following their hospital admission. “Control clinic” group comprising 10 horses referred to the clinic for non‐gastrointestinal abnormalities (such as ovariectomy, castration, wound treatment, or orthopedic procedures) formed the group. In this group, hematology was tested, excluding lactate levels. The “control stable” group comprised eight healthy horses, kept under identical conditions without location changes for the past 3 months and were not transported. No blood tests were conducted on the “control stable” group horses. Horses diagnosed with colic were treated pharmacologically or surgically in accordance with the art of veterinary medicine. A detailed description of the procedures is included in Supporting Information S1. Horses within the control clinic group underwent diverse procedures upon admission, in accordance with diagnostic and therapeutic protocols, inclusive of general anesthesia. It is noteworthy that none of these procedures pertained to the gastrointestinal tract. Conversely, the control stable group was exempted from any procedural interventions and remained within their designated living quarters.

### 2.2 Samples Collection

Upon admission, fecal samples were obtained (D0) and subsequently collected at 24‐h intervals for six consecutive days (D1, D2, D3, D4, D5, D6). Collection ceased sooner if the horse was discharged or euthanized as a result of severe colic. Samples came directly from the rectum during rectal exams (if it was necessary to perform) or from the bedding (directly after spontaneous defecation), selecting the central part of the fecal mass. According to the authors' experience, the way the stool sample was collected from the rectum or after spontaneous defecation did not affect the values obtained. All samples were stored at −20°C.

### 2.3 Sample Analysis

#### 2.3.1 Fecal SIgA Analysis

The analysis of fecal SIgA across all samples was conducted at the serology laboratory of the Department of Immunology, Pathophysiology, and Veterinary Preventive Medicine at Wroclaw University of Environmental and Life Sciences, employing a previously documented methodology with modifications [12, 13]. The concentration of SIgA in fecal samples was quantified utilizing a sandwich ELISA assay according to previous description [13]. All samples and calibrator were analyzed in duplicate. The intra‐assay coefficient of variation (CV) for SIgA concentrations in fecal samples was 2.7%, while the inter‐assay CV was 9.5%. The CV was estimated from the concentration of SIgA (ng/mL) in a selected fecal sample. The results were presented in units of μg/g of feces.

#### 2.3.2 D/L‐Lactate Concentration in Fecal Samples

The concentration of D/L‐lactate in fecal samples (samples D0 from all horses) was assessed utilizing the D/L‐lactic acid kit (BioSenTec, Portet‐sur‐Garonne, France; Cat. no. 023) following the manufacturer's guidelines with modifications implemented by our research team. The reagent volumes were decreased tenfold. Optical density was determined at a wavelength of 340 nm employing path length correction using the μQuantum Microplate Reader (BioTek Instruments, Vermont, USA). The analysis was conducted on a 96‐well UV‐Star microplate, F‐bottom (Greiner Bio‐One GmbH, Kremsmünster, Austria). All samples underwent analysis in duplicate. Initial reagents, specifically R1, R2, R3, along with Milli‐Q water, were mixed in suitable volumes, and subsequently, 212 μL were dispensed per well. An additional 10 μL of Milli‐Q water (blank), samples, or control solution (positive control) was incorporated per well. The reaction was initiated by the addition of 2 μL of the R4 reagent, followed by the addition of the R5 reagent. For instances where the D/L‐lactate concentration exceeded 0.2 g/L, the samples were subjected to dilution with water at ratios of either 1:2 or 1:4. The D‐lactate control exhibited a value of 0.101 ± 0.002 g/L, while the L‐lactate control displayed a value of 0.101 ± 0.003 g/L. The results, originally in g/L, were converted to mmol/L, based on a molar mass assumption of 90.8 g/mol for D‐lactate and 89.07 g/mol for L‐lactate.

### 2.4 Statistics

The statistical analysis was conducted using Python version 3.8.15, with libraries such as NumPy version 1.23.5, Pandas version 2.0.3, SciPy version 1.10.1 for statistical tests, and Statsmodels version 0.13.2 for mixed‐effects modeling. Data visualizations were crafted using Matplotlib version 3.6.2 and Seaborn version 0.12.2. To evaluate differences between two independent groups, the Mann–Whitney *U* test was utilized. For analyzing more than two groups, the Kruskal–Wallis test was used. After significant differences were found with the Kruskal–Wallis test, the Mann–Whitney *U* test was used for post hoc pairwise comparisons between specific group pairs. For longitudinal data analysis, a linear mixed‐effects model was applied through the Statsmodels library, which facilitated the examination of SIgA level changes across groups over time. This model included both fixed (group and time) and random effects, with the control stable group taken as the reference. The model analyzed initial SIgA levels (intercepts) and their changes over time (slopes) for each group. Spearman's rank correlation was used for non‐normally distributed data, and Pearson's correlation for normally distributed data. All statistical tests were two‐tailed with a significance level of *p* < 0.05. The analysis provided both summary statistics and significance tests to support the interpretation of the data. Boxplots were used to visualize distributions, with median and interquartile ranges indicated for each group. In order to control the global Type I error rate across the entire manuscript, the Bonferroni correction was applied to *p* values. Because we performed 19 statistical tests, however 11 of them are presented in this study, each resulting *p* value was multiplied by 19. In post hoc tests, the Bonferroni correction was also used, taking into account the specific number of pairwise comparisons performed in each respective analysis. The generalized linear model, which describes SIgA concentration over time, was not subjected to Bonferroni correction. Furthermore, to evaluate effect sizes, we calculated Cliff's delta comparisons that were statistically significant. We employed a generalized linear model (GLM) with day and group as fixed effects, along with their interaction, to evaluate log10‐transformed SIgA levels over the course on seven following days. This model allowed us to assess changes in SIgA levels over time across multiple groups. It accounted for fixed effects (group and time) with the control stable group set as the reference category. The model evaluated baseline SIgA levels (intercepts) and their rate of change over time (slopes) for each group. We have used Holm's correction to control for the false positives.

## 3 Results

### 3.1 Animals

Seventy horses met the criteria for inclusion in the study which resulted in 16 horses in colic surgical group, 36 horses in colic medical group, 10 horses in control clinic group, and eight horses in control stable group. In three horses from colic surgical group, the owners declined the surgical procedure due to financial constraints, resulting in euthanasia of the horses. Another four horses from colic surgical group were euthanized during laparotomy procedure due to grave prognosis. Thus, only nine out of 16 horses from the colic surgical group recovered after surgery and were available for sample collection during hospitalization. Of all colic cases, 12 out of 52 were associated with lesion of the small intestine, predominantly involving small intestinal strangulation (six cases), suspected ileum impaction (five cases), and enteritis (one case). In contrast, 40 out of 52 cases entailed lesions of the large intestine, such as large colon impaction (19 cases), large colon displacement (11 cases), large colon torsion (two cases), small colon impaction (two cases), caecum impaction (two cases), large colon mass resection (one case), and mild colics with no definitive diagnosis (three cases). Among horses in the colic surgical group, 8/16 horses had strangulating lesions or torsion, which are considered a strong cause of intestinal ischemia. Among these horses, all showed elevated serum lactate concentration, and three also had elevated lactate levels in the peritoneal fluid. The results of basic laboratory test are described in Table 1.

**TABLE 1:** Laboratory variables in tested horses.

Parameter	Colic medical	Colic surgical	Control clinic	Normal range
WBC [×10^3^/μL]	Median 7.44 (Q1 = 6.00, Q3 = 9.55)	Median 8.40 (Q1 = 6.50, Q3 = 9.20)	Median 7.89 (Q1 = 6.47, Q3 = 9.12)	10.1 × 10^3^/μL
PCV	Median 0.33 (Q1 = 0.298, Q3 = 0.35)	Median 0.3 (Q1 = 0.285, Q3 = 0.365)	Median 0.325 (Q1 = 0.293, Q3 = 0.378)	0.30–0.50
Total protein [g/L]	Median 64.3 (Q1 = 59.0, Q3 = 65.8)	Median 63.5 (Q1 = 59.6, Q3 = 71.8)	Median 63.5 (Q1 = 60.7, Q3 = 67.5)	53–73 g/L
Blood lactate [mmol/L]	Median 1.1 (Q1 = 0.9, Q3 = 2.7)	Median 1.4 (Q1 = 0.75, Q3 = 3.7)	nd	< 2 mmol/L
Peritoneal lactate [mmol/L]	Median 1.4 (Q1 = 0.8, Q3 = 2.3)	Median 2.0 (Q1 = 1.2, Q3 = 4.7)	nd	< 2 mmol/L

Abbreviations: nd, not detected; WBC, white blood cells.

### 3.2 Fecal SIgA Concentration

**FIGURE 1:**
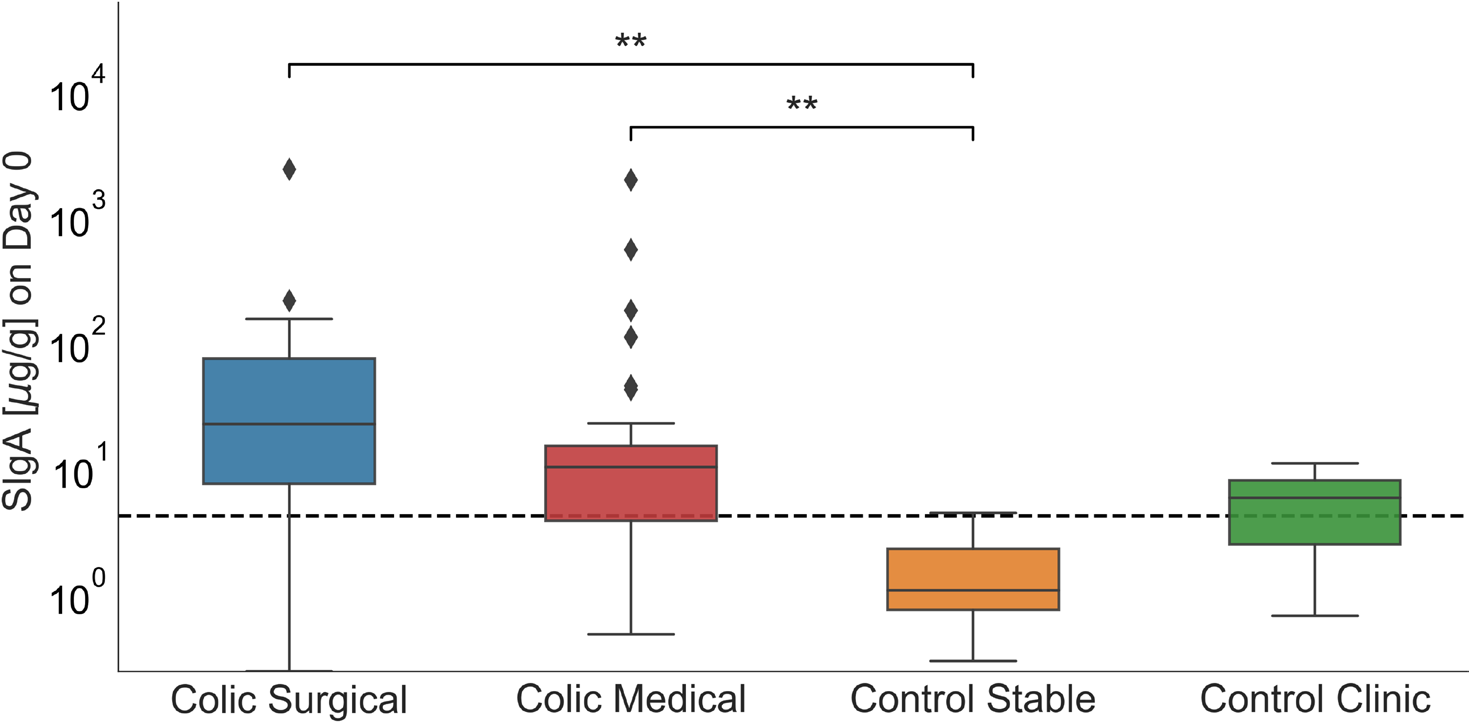
Distribution of SIgA concentrations on Day 0 across four groups (colic medical, colic surgical, control stable, control clinic). **p < 0.01.

The results of SIgA concentration on Day 0 are presented on Figure 1. The analysis showed no significant difference between SIgA values at time D0 between the colic medical and colic surgical groups, and between control clinic and control stable group. Values in the colic medical and colic surgical groups were significantly higher than in the control stable group (*U* = 126.0, *p* = 0.099, Cliff's ∆ = 0.58 and *U* = 248.0, *p* = 0.005, Cliff's ∆ = 0.72, respectively). Detailed value of fecal SIgA concentration in different groups were presented in Table 2.

**TABLE 2:** Fecal SIgA and lactate concentration in different groups in D0.

Parameter	Colic medical	Colic surgical	Control clinic	Control stable
SIgA [μg/g]	Median 10.03 (Q1 = 3.77, Q3 = 13.83, IQR = 11.06)	Median 22.09 (Q1 = 7.42, Q3 = 72.72, IQR = 65.3)	Median 5.73 (Q1 = 2.43, Q3 = 7.87, IQR = 5.44)	Median 1.06 (Q1 = 0.74, Q3 = 2.25, IQR = 1.51)
L‐lactate [mmol/L]	Median 0.59 (Q1 = 0.36, Q3 = 0.72, IQR = 0.36)	Median 0.65 (Q1 = 0.46, Q3 = 1.01, IQR = 0.55)	Median 0.97 (Q1 = 0.77, Q3 = 1.27, IQR = 0.50)	Median 1.01 (Q1 = 0.77, Q3 = 1.17, IQR = 0.4)
D‐lactate [mmol/L]	Median 1.08 (Q1 = 0.48, Q3 = 1.42, IQR = 0.94)	Median 0.97 (Q1 = 0.73, Q3 = 2.31, IQR = 1.58)	Median 1.52 (Q1 = 1.21, Q3 = 1.92, IQR = 0.71)	Median 2.06 (Q1 = 1.54, Q3 = 2.96, IQR = 1.42)

Abbreviation: IQR, interquartile range.

**FIGURE 2:**
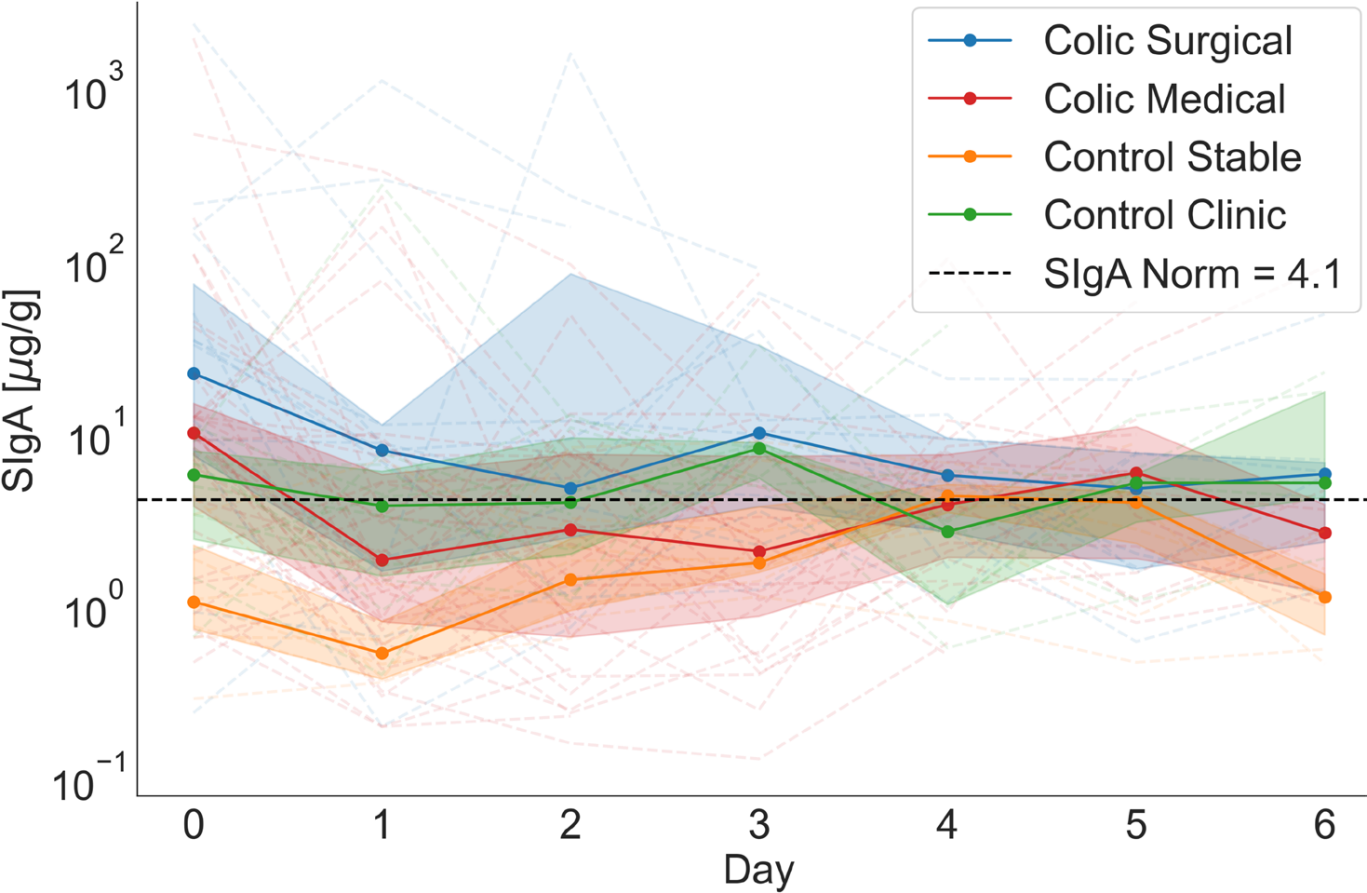
Secretory IgA concentrations over time in four groups: colic surgical, colic medical, control stable, and control clinic. The thick lines represent the median SIgA levels for each group across time points (Day 0 to Day 6). Individual values for each subject are shown as thin dashed lines. The shaded areas represent the interquartile range, spanning from the 25th to the 75th percentile.

A mixed‐effects linear model was employed to examine the variations in fecal SIgA levels over time across four distinct groups of horses (Figure 2). The dependent variable, log10‐transformed IgA concentrations, was assessed longitudinally (Day 0–Day 6). The model incorporated the effects of the groups (control clinic, colic medical, colic surgical, and control stable) and time as covariates, in addition to their interactions. The intercept for control stable did not significantly differ from zero (*p* = 0.828), indicating that the baseline log10(SIgA) levels in this group are not significantly different from zero at Day 0. The intercept for control clinic was not significantly different from control stable (*p* = 0.160), indicating that the control clinic group has comparable baseline log10(SIgA) levels compared to control stable at Day 0. The intercept for colic medical and colic surgical was significantly higher than control stable (*p* = 0.0021 and *p* = 0.008, respectively), suggesting that horses in both colic groups had higher baseline log10(SIgA) levels compared to the control stable group. The slope for control clinic did not significantly differ from control stable (*p* = 0.828), suggesting that log10(IgA) levels in the control clinic group also remain stable over time. The slope for colic medical and colic surgical was significantly different from control stable (*p* = 0.021 and *p* = 0.040, respectively), indicating a significant decrease in log10(SIgA) levels over time in both colic groups. In summary, the model shows that while the control stable and control clinic groups had a consistent baseline and stable levels of immunoglobulin A over time, the colic medical and colic surgical groups had significantly higher baseline IgA levels. Both the medical and surgical groups experienced significant decreases in log10(IgA) levels over time compared to control stable, with the surgical group showing the largest decrease. control clinic group had stable level of SIgA over time.

As we wanted to assess differences in SIgA concentrations based on the outcome due to the resolution of the colic condition, whether through euthanasia or patient recovery, fecal SIgA concentrations at Day 0 were also evaluated within the “clinic” group (comprising all horses, both clinic surgical and clinic medical). The results of this test indicate that there is no significant difference in SIgA concentrations attributable to the recovery outcome, as suggested (Mann–Whitney *U* test, yielding a *U* statistic of 105.0 with a *p* value of 0.167).

### 3.3 Fecal D‐Lactate and L‐Lactate Concentrations (Mmol/L)

**FIGURE 3:**
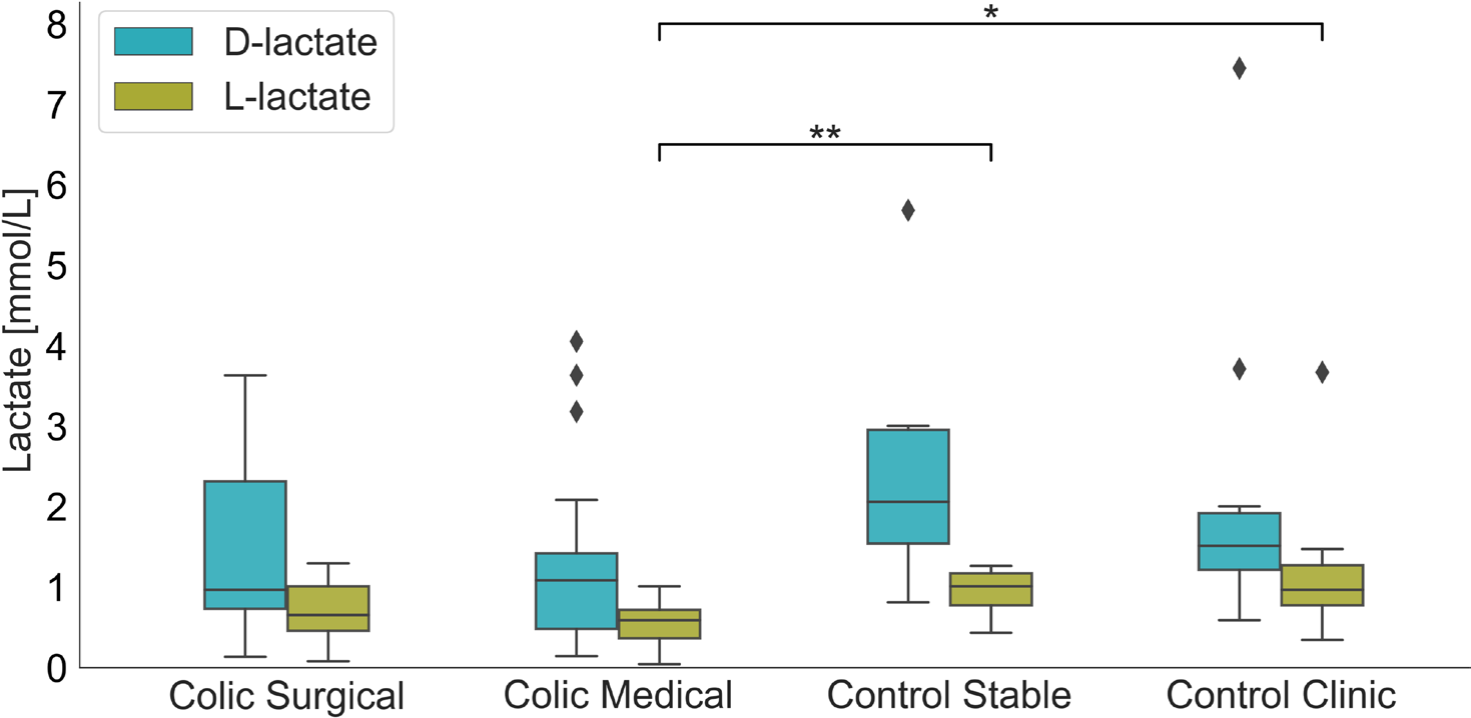
Distribution of D‐lactate and L‐lactate concentrations (mmol/L) across four groups (colic surgical, colic medical, control stable, control clinic). The blue boxplots represent D‐lactate, and the green boxplots represent L‐lactate. Kruskal–Wallis tests revealed significant differences among the groups for L‐lactate (*H* = 15.61, *p* = 0.019). Post hoc Mann–Whitney *U* tests showed significant for L‐lactate between the colic medical and control stable groups (*U* = 38.5, *p* = 0.010, Cliff's ∆ = −0.72) and between the colic medical and control clinic groups (*U* = 62.0, *p* = 0.013, Cliff's ∆ = −0.65). Statistical significance levels are indicated by asterisks as follows: *p < 0.05, **p < 0.01.

The results of fecal D‐lactate and L‐lactate concentrations are presented on Figure 3. Kruskal–Wallis tests revealed significant differences among the groups for L‐lactate (*H* = 15.61, *p* = 0.019). Post hoc Mann–Whitney *U* tests showed significant for L‐lactate between the colic medical and control stable groups (*U* = 38.5, *p* = 0.010, Cliff's ∆ = −0.72) and between the colic medical and control clinic groups (*U* = 62.0, *p* = 0.013, Cliff's ∆ = −0.65). There were lack of differences in L‐lactate levels in colic surgical groups. Detailed value of fecal lactate concentration in D0 in different groups were presented in Table 2.

### 3.4 SIgA Correlation With Inflammatory Status/Lactates

**FIGURE 4:**
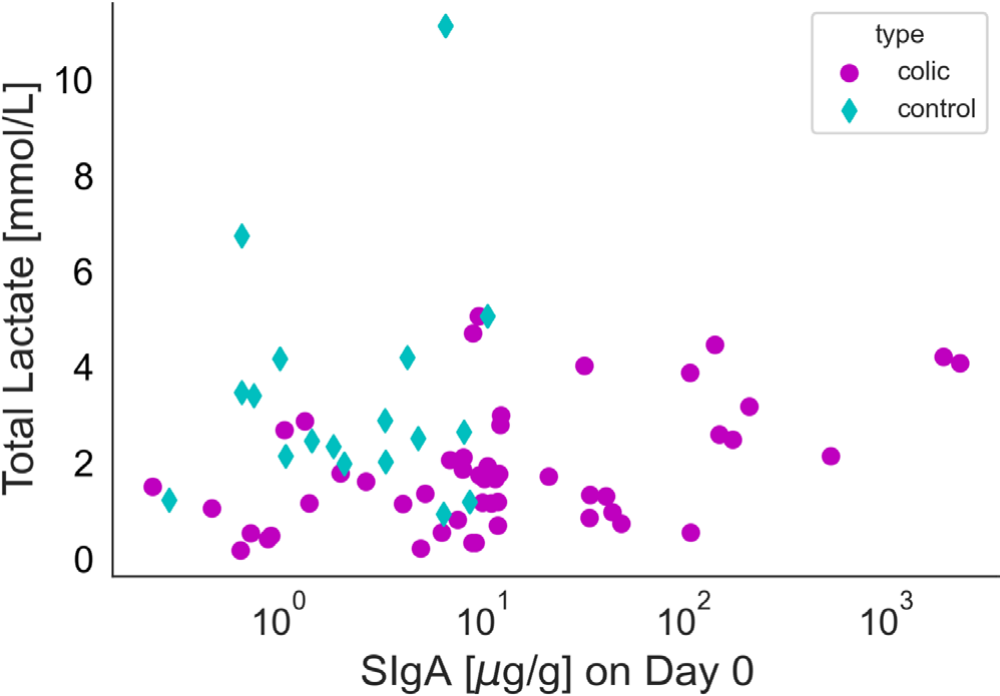
Correlation between fecal secretory IgA and total lactate concentration in colic and control horses. Spearman correlation coefficient = 0.421, *p* = 0.038 (significant at *p* < 0.05).

Spearman and Pearson correlation analyses were conducted to evaluate the relationship between “SIgA in Day 0” and several parameters (“WBC,” “total protein,” “total fecal lactate concentration”) within two groups: “colic” (colic medical + colic surgical) and “control” (control clinic + control stable). Within the “colic” group, the Spearman correlation was employed to determine the association between SIgA in Day 0 and D‐ and L‐lactate sum, resulting in a Spearman correlation coefficient of 0.421, with *p* = 0.038 (Figure 4). No other correlations were observed.

## 4 Discussion

Horses with colic had notably higher fecal SIgA levels compared to the control groups. The results demonstrated that horses examined at the clinic for non‐gastrointestinal abnormalities also had higher fecal SIgA, but to a much lesser extent than those with colic. This indicates a possible effect of stress and transport on SIgA values however, to a much lower degree than colic [11]. The study did not determine the effectiveness of fecal SIgA levels as a prognostic marker or their usefulness in guiding treatment choices, whether conservative or surgical.

Strangulating obstruction is a condition where the blockage of both the intestinal lumen and blood vessels leads to ischemic damage to the mucosa, and it is a leading cause of death related to colic in horses [1]. However, horses with other types of intestinal disorders besides strangulating obstruction can also experience ischemic conditions. In cases of simple obstruction, the pressure in the intestinal lumen can increase significantly, which can also result in ischemia and ultimately ischemic necrosis of the intestine [2]. The results of the study showed an increase in fecal SIgA during colic, which might be attributed to the onset of an inflammatory state and the requirement to enhance mucosal defenses. This could also be due to a considerable slowing of peristalsis, leading to prolonged contact between feces and the intestinal mucosal surface. These findings are contrary to data from mice wherein experimental intestinal ischemia was induced by blocking the superior mesenteric artery [7]. Intestinal ischemia resulted in a reduction in IgA+ cells (responsible for IgA production in the lamina propria), a decrease in IgA mRNA expression in the intestinal lining, and a lowered SIgA concentration in the intestinal fluid [7]. However, in the study mentioned above, the entire intestine was ischemic, unlike in colic horses, where ischemic changes begin locally at the site of fecal blockage or intestinal twisting. The authors believe that in horses with colic, sections of the intestine not affected by ischemia assume a compensatory role, increasing the secretion of SIgA by IECs. Nevertheless, the absence of concurrent immunohistochemical analysis of inflamed intestinal sections means that it is unclear whether there is an enhanced transport of SIgA or an increase in the number of IgA+ cells and its overproduction in the lamina propria due to inflammation and cytokine chemotactic effects. Despite a range of studies conducted on mice and humans, some mechanisms controlling SIgA functions remain unidentified [14]. Therefore, it can be hypothesized that significantly elevated SIgA levels in the feces of colic horses indicate inflammation. However, no statistically significant differences were observed in SIgA levels between horses with varying colic severity and treated via different methods. Additionally, no differences in fecal SIgA were noted concerning prognosis (survivors compared to non‐survivors). Among the many available markers of inflammation and intestinal ischemia, the study presented here assessed WBC and lactate levels in serum and peritoneal fluid. However, there was no correlation between SIgA and the mentioned variables in the studied horses.

The study's second objective was to evaluate the changes in fecal SIgA levels during the treatment phase, considering both conservative approaches (such as fluid therapy, pharmacotherapy, and diet therapy) and surgical interventions. The analysis provided here indicates a notable drop in SIgA during treatment for both the colic medical and colic surgical groups. Nonetheless, these are initial observations that do not consider the length of treatment and other elements that might influence the parameter being examined, so conclusions should be approached with care. Horses in both colic medical and colic surgical categories underwent pharmacological treatments involving spasmolytic and analgesic medications. The typical approach for treating colicky horses includes administering scopolamine butylbromide via intravenous injection, a drug known for its smooth muscle relaxant properties [15]. Research indicates that through antimuscarinic action, the analogous compound hyoscine butylbromide diminishes the secretory activity of IECs, including mucus secretion [16]. It has no beneficial impact on intestinal healing processes [17]. However, the effect of spasmolytic drugs on SIgA secretion in the gastrointestinal tract in both humans and horses remains uncertain. To restore barrier function, it is essential to reestablish the tight junctions between cells, which can be compromised by the influx of inflammatory cells and the nonsteroidal anti‐inflammatory drugs (NSAIDs) administered to treat colic in horses [18, 19]. During the healing phase, epithelial reformation occurs using intestinal stem cells, which might take 3–7 days to differentiate [1]. NSAIDs' analgesic and anti‐inflammatory mechanisms involve the inhibition of the cyclooxygenase enzyme and prostaglandin production [20]. Despite their benefits, this mechanism could potentially harm the natural protective barriers of the gastrointestinal mucosa. A study by Adam et al. 2017 found that administering two NSAIDs, metamizole and flunixin meglumine, to horses significantly increased urinary SIgA secretion, although its impact on intestinal SIgA production is still unknown [21]. Metamizole predominantly impairs the healing of muscular rather than mucosal membranes in intestinal structures [22]. Opioids like morphine are also employed in colic pain management [20]. In mice, morphine did not alter the total SIgA production by intestinal cells, but it lowered the production of SIgA specific to the tested antigen and influenced mucosal morphological changes, potentially impacting repair processes [23, 24]. Nonetheless, the question of how analgesic therapy influences the SIgA levels in horse feces requires further exploration. Treating horses with colic might also necessitate broad‐spectrum antimicrobials like penicillin and gentamicin, especially for those undergoing surgery or requiring transabdominal or transrectal trocarisation, or both. Research in humans and mice suggests the potential for developing secondary IgA deficiency in the lungs as a negative outcome of antimicrobial therapy [25]. However, similar information for horses is lacking. In brief, different medications might impact SIgA secretion in the gastrointestinal tract in various ways; yet, their primary purpose is to address the causes of colic (such as smooth muscle contraction) and to alleviate pain and inflammation simultaneously. This study demonstrated that properly administered conservative treatment in mild colic cases resulted in both recovery and a reduction in fecal SIgA levels. Surgical treatment also required general anesthesia. Research on horses has demonstrated that xylazine (from the a‐2‐agonist category) used for premedication has a positive effect on mucosal inflammation in horses with colic [26]. Lidocaine administered preoperatively has no effect [26]. General anesthesia led to a noticeable decrease in fecal SIgA in tested horses, irrespective of their medical condition type (also without surgery) [10]. In rats, general anesthesia induced by ketamine is linked to reduced intestinal injury and prevents the intestinal transit delay caused by ischemia/reperfusion [27]. However, in horses, the influence of isoflurane anesthesia on intestinal tissue oxygenation and blood flow has also been evaluated [28]. With isoflurane, intestinal perfusion remained intact or blood flow limit was reached, representing a dose‐dependent effect rather than related to anesthesia duration. Surgical operations can adversely affect fecal SIgA concentrations in horses, indicating an increased risk of postoperative infections [10]. Surgical intervention for acute colic, as highlighted by the comprehensive longitudinal analysis, results in a significant and marked reduction of fecal SIgA levels. This research found that a postoperative decrease in SIgA seems favorable for colic horses, implying a reduction in existing inflammation.

Transporting horses to the clinic and their subsequent hospitalization reduced fecal SIgA levels [10]. These findings contrast with the current study, where the control clinic group exhibit higher fecal SIgA levels compared to the control stable group. Hospitalization in the control clinic group did not lead to substantial changes in fecal SIgA during the course of study. These discrepancies could be due to methodological variations. Nonetheless, in a research study, one horse from the “gastrointestinal disorders” category, presenting with colon torsion, showed higher fecal SIgA levels [10]. Although the sample size was too small to determine the significance of this occurrence, it supports the findings of the study presented here.

Currently, only a limited number of studies examine D‐ and L‐lactate in fecal samples within human medicine, and the findings are associated with dysbiosis in individuals suffering from colitis and short bowel syndrome [29, 30, 31]. Until now, only increased levels of peritoneal fluid and serum lactate have been acknowledged in diagnosing colic horses, with a particular focus on L‐lactate as indicators of tissue hypoxia [5]. The research highlighted in this study is the inaugural attempt to quantitatively evaluate D‐ and L‐lactate in the feces of horses with colic. Fecal D‐ and L‐lactate values in the study groups were higher than in healthy horses in studies available in the literature [32]. This might be due to the different methods used for the analysis and the lack of reference standards for horses. It is proposed that lactic acid producing bacteria might also contribute to immune system activation, although no link between lactic acid bacteria, lactic acid production, and SIgA has been identified in healthy horses [12]. Lactic acid accumulation can potentially irritate or harm the intestinal lining and might modify the permeability of the large intestine's mucosa to toxins and larger molecules, which have been implicated in laminitis development [33]. This study found that horses in the colic medical group exhibited significantly lower L‐lactate levels in their feces compared to controls; nevertheless, the D/L ratio was stable, eliminating the possibility of bacterial overgrowth and excess D form production. Existing literature suggests that lactic acid bacteria like *Lachnospiraceae* and *Lactobacillaceae* were prevalent in horses with large intestinal colic [34]. However, how this affects lactate production and its detectability in feces remains largely unknown. The discovered correlation between SIgA levels and total lactate concentration in colic horses (considering both types) appears intriguing and suspected to be related to ischemic disorders.

Clinically, the potential application of the described method for forecasting the progression of colic is significant in this study. Currently, the study only provided a definitive result regarding the rise in fecal SIgA in colic horses, but the sample size was insufficient to establish reference values as well as the method's sensitivity and specificity. Performing an ELISA test is simple and relatively affordable; however, it necessitates specialized equipment for result interpretation, making it unsuitable for clinical use at the moment.

The research encompassed a limited number of cases and did not distinguish between those with strangulation disorders and non‐strangulation.

In conclusion, the research presented here demonstrates the feasibility of using a fecal sample for biomarker analysis. Colic causes a significant increase in fecal SIgA concentration. A correlation has emerged between fecal SIgA concentration and total fecal lactate concentration, which might be related to the pathogenesis of ischemic disorders. The comprehensive data collected underscore the profound dependence of colic disorders on both barrier and immune functions of the gastrointestinal tract, along with transformative changes in the metabolic activity of the intestinal microbiota.

## Disclosure

Authors declare no off‐label use of antimicrobials.

## Ethics Statement

Approved by the Ethics Committee of the University of Veterinary Sciences Brno, protocol number:ES_2‐2024. Authors declare human ethics approval was not needed.

## Conflicts of Interest

The authors declare no conflicts of interest.

## Supporting information


Data S1.
