# Supplementary material for: Fecal Secretory Immunoglobulin A and Lactate Level as a Biomarker of Mucosal Immune Dysfunction in Horses With Colic
Source: J Vet Intern Med. 2025 Mar 27;39(3):e70073. doi: 10.1111/jvim.70073 (PMC11947735; doi:10.1111/jvim.70073)
Supplement: Supplementary file 1 — Data S1. [file JVIM-39-e70073-s001.docx]

**Supplementary Material 1**

*Surgical colic treatment procedure - laparotomy:*

An exploratory laparotomy was executed utilizing the conventional ventral midline approach under general anesthesia, with horses positioned in dorsal recumbency. Horses were premedicated with antibiotics, specifically potassium penicillin G at a dosage of 22,000 IU/kg intravenously (Pencilin G draselná sůl BIOTIKA, BB pharma a.s., Czech Republic) and gentamicin at 6.6 mg/kg intravenously (Aagent; Fatro S.p.A., Italy), administered within one hour to ten minutes preceding the induction of anesthesia. Flunixin meglumine at 1.1 mg/kg intravenously (Flunbix; Fatro S.p.A., Italy) was additionally utilized as premedication, contingent upon its absence during colic treatment within ten hours prior to surgical intervention. Immediately preceding anesthesia, premedication with xylazine at 1.1 mg/kg intravenously (Xylazin Ecuphar; Riemser Arzneimittel AG, Germany) was administered. Anesthesia induction occurred five minutes thereafter using ketamine at 2.2 mg/kg intravenously (Narkamon 10%; Bioveta a.s., Czech Republic) in conjunction with diazepam at 0.02 mg/kg intravenously (Apaurin; Krka d.d., Slovenia). Maintenance of anesthesia was achieved with isoflurane (Aeranne; Baxter S.A., Belgium) vaporized within oxygen. Postoperative management comprised a continuous rate infusion (CRI) of lidocaine at 1.3 mg/kg bolus; 3 mg/kg/h CRI (Lidocaini Hydrochloridum 25g pulv.; Pharmacy Sv. Anna, Czech Republic) over 12-24 hours, fluid therapy spanning 2-5 days, antibiotic regimen (potassium penicillin G 22,000 IU/kg intravenously QID (Pencilin G draselná sůl BIOTIKA, BB pharma a.s., Czech Republic); gentamicin 6.6 mg/kg intravenously SID (Aagent; Fatro S.p.A., Italy)) for 3-5 days, and administration of flunixin meglumine 1.1 mg/kg intravenously SID-BID (Flunbix; Fatro S.p.A., Italy) for 2-5 days. A subset of horses additionally received treatment with polymyxin 1,000-6,000 IU/kg intravenously in three doses every 12 hours (Polymixin B sulfate 50-500 mg; Pharmacy Sv. Anna, Czech Republic), and/or enoxaparin 40 IU/kg subcutaneously once a day (Clexane 2000-15000 IU; Sanofi-Aventis, France), contingent upon systemic disease presence and endotoxemia. If gastric reflux was observed, stomach evacuation via nasogastric tube was routinely performed. The dietary reintroduction involved initial administration of soaked hay pellets, subsequently transitioning to hay, administered gradually over a period of 3-5 days for patients undergoing major intestinal surgery, and extending to 5-7 days for those undergoing minor intestinal interventions.

*Conservative colic treatment procedure:*

Medical treatment of colic patients consisted of analgesic treatment to effect (flunixin meglumine 1.1 mg/kg i.v. (Flunbix; Fatro S.p.A., Italy), metamizole 20-50 mg/kg i.v. (Vetalgin, MSD Intervet, Netherlands), detomidine 5-20 µg/kg i.v. (Cepesedan; Cp-pharma Handelsgesellschaft mbH, Germany), xylazine 0.4-0.7 mg/kg i.v. (Xylazin Ecuphar; Bioveta a.s., Czech Republic), butorphanol 0.01-0.04 mg/kg i.v. (Butomidor, Richter Pharma AG, Austria), morphine 0.05-0.1 mg/kg i.v. (Morphin BIOTIKA 1%; BB pharma a.s., Czech Republic)), spasmolytics (butylscopolamine 0.2-0.3 mg/kg i.v. (Buscopan; Boehringer Ingelheim International GmbH, Germany)), parenteral and peroral fluid therapy, and laxatives (mineral salts). The use of drugs, length and intensity of the treatment varied considerably with diagnoses. A definitive diagnosis could not always be established. Most simple impactions of the large colon were resolved within one to three days of treatment. If the horse showed severe tympany of the large intestine, transabdominal and/or transrectal trocarization was performed for intestinal decompression, and horses were treated with antibiotics (potassium penicillin G 22 000 IU/kg i.v. QID (Pencilin G draselná sůl BIOTIKA, BB pharma a.s., Czech Republic); gentamicin 6.6 mg/kg i.v. SID (Aagent; Fatro S.p.A., Italy)) for one to three days to reduce the risk of secondary peritonitis. Feed was slowly introduced over three to five days as soon as the horse showed no signs of colic, had a good appetite, and the rectal examination was without abnormal findings.
